# GCN5 contributes to stem cuticular wax biosynthesis by histone acetylation of *CER3* in Arabidopsis

**DOI:** 10.1093/jxb/ery077

**Published:** 2018-03-01

**Authors:** Tianya Wang, Jiewen Xing, Xinye Liu, Yingyin Yao, Zhaorong Hu, Huiru Peng, Mingming Xin, Dao-Xiu Zhou, Yirong Zhang, Zhongfu Ni

**Affiliations:** 1 Key Laboratory of Molecular Epigenetics of the Ministry of Education (MOE), Northeast Normal University, Changchun, China; 2 State Key Laboratory of Agrobiotechnology, Key Laboratory of Crop Heterosis and Utilization (MOE), Key Laboratory of Crop Genetic Improvement (Beijing Municipality), China Agricultural University, Beijing, China; 3 College of Agronomy and Biotechnology, China Agricultural University, Beijing, China; 4 Institute of Plant Science Paris-Saclay, Université Paris Sud, 91405 Orsay, France; 5 National Maize Improvement Center, China Agricultural University, Beijing, China

**Keywords:** *Arabidopsis thaliana*, *CER3*, GCN5, histone acetylation, stem, wax biosynthesis

## Abstract

Cuticular wax is a major component of the surface cuticle of plants, which performs crucial functions in optimizing plant growth. Histone acetylation regulates gene expression in diverse biological processes, but its role in cuticular wax synthesis is not well understood. In this study, we observed that mutations of the *Arabidopsis thaliana* histone acetyltransferase GENERAL CONTROL NON-REPRESSED PROTEIN5 (GCN5) impaired the accumulation of stem cuticular wax. Three target genes of GCN5, *ECERIFERUM3* (*CER3*), *CER26*, and *CER1-LIKE1* (*CER1-L1*), were identified by RNA-seq and ChIP assays. H3K9/14 acetylation levels at the promoter regions of *CER3*, *CER26*, and *CER1-L1* were consistently and significantly decreased in the *gcn5-2* mutant as compared to the wild-type. Notably, overexpression of *CER3* in the *gcn5-2* mutant rescued the defect in stem cuticular wax biosynthesis. Collectively, these data demonstrate that GCN5 is involved in stem cuticular wax accumulation by modulating *CER3* expression via H3K9/14 acetylation, which underlines the important role of histone acetylation in cuticular wax biosynthesis.

## Teaser

The histone acetyltransferase GCN5 modulates stem cuticular wax accumulation by acetylating the key wax biosynthetic enzyme gene *CER3* in Arabidopsis.

## Introduction

Plant cuticular wax is a complex mixture of very-long-chain fatty acids (VLCFAs) and aldehydes, alcohols, alkanes, ketones, and esters, with predominant carbon chain-lengths ranging from C22 to C36 (Samuels *et al.*, 2008; Li *et al.*, 2016), and it forms one of the major lipid components of the cuticle that covers the outer surface of aerial plant tissues. The biosynthesis of cuticular wax is processed through two distinct pathways, termed the alcohol-forming and the alkane-forming pathways, which yield 17~18% and 80% of the total amount of wax, respectively (Bernard and Joubès, 2013). As a crucial adaptive characteristic, cuticular wax protects plants against biotic and abiotic stresses, such as pathogen attacks and water loss (Aharoni *et al.*, 2004; Samuels *et al.*, 2008). Therefore, elucidating the regulatory mechanisms controlling cuticular wax accumulation is of great interest for genetic engineering of agricultural crops.

To date, numerous candidate genes in the cuticular wax pathway have been identified, most of which encode enzymes, or work with enzymes, in the VLCFA biosynthesis and derivatization pathways (Koornneef *et al.*, 1989; Bernard and Joubès, 2013). For example, CER2, and its homologues CER2-LIKE1/CER26 and CER2-LIKE2 have biological functions in two-carbon elongation of VLCFAs, from C28 to C30, C30 to C32, and C32 to C34, respectively (Haslam *et al.*, 2012, 2015; Pascal *et al.*, 2013; Lee and Suh, 2015). A member of the bifunctional wax ester synthase/diacylglycerol acyltransferase family, WSD1, is a key enzyme in wax ester synthesis of Arabidopsis stems (Li *et al.*, 2008). As for the essential alkane-forming pathway, *CER3/WAX2* plays an important role in synthesis of major wax components (Aarts *et al.*, 1995; Chen *et al.*, 2003; Rowland *et al.*, 2007; Lee and Suh, 2015). The amount of wax found in the *cer3* mutant was severely reduced compared with the wild-type in Arabidopsis stems, especially with regards to aldehydes, alkanes, secondary alcohols, and ketones (Rowland *et al.*, 2007). Although the exact reactions catalysed by the CER3 enzyme remain unknown, it has been reported that CER3 may physically interact with CER1 and CYTOCHROME B5 ISOFORM (CYTB5) for the biosynthesis of very-long-chain alkanes (Bernard *et al.*, 2012).

An increasing focus on the transcriptional regulation of the genes in cuticular wax biosynthesis has led to some transcription factors being reported in recent studies (Lee and Suh, 2015). In Arabidopsis, WAX INDUCER1/SHINE1 (WIN1/SHN1), an AP2-EREBP-type transcription factor, was the first to be identified and is a representative regulator of wax biosynthesis, regulating the *CER1*, *CER2*, and *3-KETOACYL-COA SYNTHASE1* (*KCS1*) genes (Aharoni *et al.*, 2004; Broun *et al.*, 2004). *WIN1* overexpression lines exhibited enhanced drought tolerance compared with the wild-type (Aharoni *et al.*, 2004). In addition, MYB transcription factors are important for wax biosynthesis under both biotic and abiotic stresses (Suh *et al.*, 2005; Lee and Suh, 2015). For example, MYB96 controls wax biosynthesis by regulating the *KCS1*, *KCS2*, *KCS6*, *BETA-KETOACYL REDUCTASE1* (*KCR1*), and *CER3* genes under drought stress (Lee *et al.*, 2016*b*), and *KCS1*, *KCR1*, *CER2*, and *CER3* are the targets of MYB30 in response to pathogen attack (Raffaele *et al.*, 2008).

With our increasing understanding of epigenetic mechanisms, recent reports have demonstrated that several epigenetic events are involved in wax biosynthesis (Lee and Suh, 2013, 2015). Recently, two RING E3 ligases, HISTONE MONOUBIQUITINATION1 (HUB1) and HUB2, were demonstrated to be involved in wax biosynthesis by monoubiquitinating histone H2B proteins, which in turn activates the transcriptional levels of the wax biosynthetic genes *LONG-CHAIN ACYL-COA SYNTHETASE2* (*LACS2*) and *CER1* (Ménard *et al.*, 2014). The Arabidopsis histone methyl transferases SET DOMAIN GROUP8 (SDG8) and SDG25 have been reported to contribute to wax accumulation through histone lysine methylation and/or H2B ubiquitination by targeting the key wax biosynthetic gene *CER3* (Lee *et al.*, 2016*a*). However, to the best of our knowledge, histone-acetylating events have not been reported in cuticular wax biosynthesis.

The reversible modulations of histone acetylation and de-acetylation are catalysed by histone acetyltransferases (HATs) and histone de-acetylases (HDACs), respectively (Pandey *et al.*, 2002). As in yeast and mammals, the Arabidopsis HATs are grouped into four classes: GNAT (GCN5-related N-acetyltransferase), MYST (for ‘MOZ, Ybf2/Sas2 and Tip60’), p300/CBP (p300/CREB-binding protein), and TAF1 [for ‘TATA-binding protein (TBP)-associated factor’] (Servet *et al.*, 2010). GCN5, a GNAT-type HAT that harbors a HAT domain and a bromodomain, has been well studied in extensive research (Benhamed *et al.*, 2008; Servet *et al.*, 2010). It has been observed that mutations of GCN5 result in various growth impairments, such as dwarfism, defects in terminal flower production, deformed seed development, and poor fertility (Bertrand *et al.*, 2003; Vlachonasios *et al.*, 2003). Moreover, GCN5 regulates a variety of biological processes in Arabidopsis, including cell differentiation, shoot and floral meristem formation, light and abiotic stress (e.g. heat and cold) responses, iron homeostasis, and fatty acid biosynthesis, by catalysing histone acetylation levels of target promoters at certain sites, including H3K14, H3K9, and H3K27 (Laux *et al.*, 1996; Stockinger *et al.*, 2001; Bertrand *et al.*, 2003; Vlachonasios *et al.*, 2003; Benhamed *et al.*, 2006; Earley *et al.*, 2007; Hu *et al.*, 2015; Xing *et al.*, 2015; Wang *et al.*, 2016). In this current study, we found that the *gcn5* mutants had significantly glossy (wax-deficient) stems compared with the wild-type. Analyses of chemical components coupled with scanning electron microscopy showed an obvious reduction in amounts of total wax and changes in their composition. Moreover, we found that GCN5 bound to the promoter of *CER3* and this interaction was impaired in the *gcn5-2* mutant. Taken together, we conclude that GCN5-mediated histone acetylation of *CER3* regulates stem cuticular wax biosynthesis in Arabidopsis.

## Materials and methods

### Plant material and growth conditions

The *Arabidopsis thaliana* wild-types Col-0 and Ws, together with T-DNA insertion mutants involved in histone modifications were used in this study, as follows. (1) Histone acetylation: *gcn5-1* and *gcn5-2* (Ws background), *hda2*, *hda2c*, *hda5*, *hda7*, *hda9*, *hda13*, *hda18*, *hda19*, and *srt2* (Col-0 background); and (2) histone methylation: *ashh1*, *ashh2*, *ashh3*, *ashh4*, *ashr2*, *ashr3*, *atx1*, *atx2*, *atx4*, *atx5*, *atxr2*, *atxr3*, and *atxr4* (Col-0 background). The *gcn5-1* and *gcn5-2* mutants were both T-DNA insertion mutants in the bromodomain-coding region (Bertrand *et al.*, 2003; Vlachonasios *et al.*, 2003; Supplementary Fig. S3A at *JXB* online). Notably, the *gcn5-2* mutation removes the entire bromodomain, which is required for binding to 11% of the GCN5 promoter targets (Servet *et al.*, 2010). The other mutants were obtained in the homozygous state from ABRC (https://abrc.osu.edu/) or from individual donors. For germination, sterilized seeds were incubated at 4 °C for 3 d, and subsequently sown on Murashige and Skoog (MS) plates containing 1% sucrose and 0.6% agar. The seedlings were grown under 16/8 h light/dark conditions at 22 °C in a growth room.

### RNA isolation and RNA-seq

Total RNA was extracted using TRIzol reagent (Invitrogen), according to the manufacturer’s instructions. RNA concentrations were measured using a NanoDrop 2000 spectrophotometer (ND-2000, ThermoFisher Scientific, Inc., MA, USA). RNA integrity was assessed using an Agilent 2100 Bioanalyser (Agilent Technologies, Inc., CA, USA). Paired-end sequencing libraries with an average insert size of 200 bp were prepared using the TruSeq RNA Sample Preparation Kit v2 (Illumina, San Diego, USA) and sequenced using a HiSeq2500 platform (Illumina, San Diego, USA) according to the manufacturers’ standard protocols. Raw data obtained from Illumina sequencing were processed and filtered using the Illumina pipeline (http://www.illumina.com) to generate FastQ files. Approximately 12 G of high-quality 125-bp paired-end reads were generated from six libraries (Supplementary Table S1). The FastQC program (http://www.bioinformatics.babraham.ac.uk/projects/fastqc/) was used to evaluate the overall quality of the RNA-seq reads. Poor-quality bases were filtered out using Sickle (https://github.com/najoshi/sickle). High-quality RNA-seq reads from each library were mapped to The Arabidopsis Information Resource (TAIR10) version of the Arabidopsis genome using the splice-junction-aware short-read alignment suite TOPHAT v2.09 with default settings (Kim and Salzberg, 2011). The reads displaying unique alignment and not more than two nucleotide mismatches were kept for further analysis. The differentially expressed genes were identified by using the edgeR package (ver. 3.2.3) with an absolute value of log_2_-fold change ≥2 and a false-discovery rate <0.05 as cut-off (Robinson *et al.*, 2010). The groups of differentially expressed genes identified by RNA-seq in this study are shown in Supplementary Table S2. Gene Ontology (GO) analysis was performed using agriGO v2.0 with a cut-off of *P*-value <0.05 (Tian *et al.*, 2017), and the total enrichment categories are identified in Supplementary Table S3.

### Quantitative real-time PCR

Real-time PCR was performed as previously described (Livak and Schmittgen, 2001) and *ACTIN8* was used as the control gene, i.e. the expression levels of each gene were normalized to that of *ACTIN8*. The primer pairs used for real-time PCR are listed in Supplementary Table S4. The PCR analysis was performed using a CFX96 System (Bio-Rad) with SYBR Green. The following program was used for the real-time PCR: 95 °C for 3 min and 40 cycles of 95 °C for 30 s, 58 °C for 30 s, and 72 °C for 30 s.

### Plasmid construction and plant transformation

A DNA fragment containing a 2.0-kb fragment upstream of the *CER3* coding sequence and full-length ORFs of *GCN5* and *CER3* were amplified by PCR-directed cloning based on the annotation from TAIR using the following primer pairs: *CER3*-P-F and *CER3*-P-R, *GCN5*-F and *GCN5*-R, *CER3*-F and *CER3*-R, respectively (Supplementary Table S4). The sequence-confirmed clones containing the ORFs of *GCN5* and *CER3* were then respectively cloned into the binary expression vector pCAMBIA1300 (driven by the CaMV35S promoter). The promoter region of *CER3* was fused to the reporter gene encoding β-glucuronidase (GUS). The chimeric gene was then cloned into the binary expression vector pCAMBIA1300 to generate *ProCER3*::*GUS*. These vectors were transferred into the *Agrobacterium tumefaciens* strain GV3101. Transgenic plants were generated using the floral dip method and subsequently screened on solid plates containing 25 mg l^–1^ hygromycin (Clough and Bent, 1998). The hygromycin-resistant seedlings were then transferred to a mixture of soil and vermiculite (2:1). At least three independent T3 homozygous lines with a single T-DNA insertion were subjected to a detailed analysis. Because the *gcn5-2* mutant exhibits low-fertility pollen that hinders the direct acquisition of transgenic plants in the *gcn5-2* background (Bertrand *et al.*, 2003; Xing *et al.*, 2015), we initially generated transgenic plants in the Ws background, and three independent transgenic T3 lines were selected for crossing into the *gcn5-2* mutant. The homologous transgenic lines in the *gcn5-2* background were selected using the same method described above.

### ChIP assay analysis

ChIP assays were performed using the Magna ChIP™ HiSens Kit (Catalogue No. 17-10460) combined with the ChIP method as previously described by Fiil *et al.* (2008). Six-week-old stems of Ws and *gcn5-2* mutants were harvested and fixed in 1% formaldehyde for 15 min in a vacuum and subsequently neutralized using 0.125 M glycine for 5 min. After washing with sterilized water, the samples were dried with towels, and ground in liquid nitrogen. The resulting powders were resuspended in the Nuclei Extraction Buffer 1, which contained 0.4 M sucrose, 10 mM Tris-HCl, pH 8.0, 10 mM MgCl_2_, 5 mM β-mercaptoethanol, 0.1 mM PMSF (Sigma, P7626), and protease inhibitors (Roche, 11873580001), and mixed immediately. After incubation for 20 min at 4 °C with a rotator, the solutions were filtered through four layers of Miracloth into new tubes, and the filtrate was then centrifuged for 20 min at 3000 *g* at 4 °C. The nuclei pellets were resuspended in Nuclei Extraction Buffer 2, which contained 0.25 M sucrose, 10 mM Tris-HCl, pH8.0, 10 mM MgCl_2_, 1% Triton X-100, 5 mM β-mercaptoethanol, 0.1 mM PMSF, and protease inhibitors. The suspensions were transferred to microfuge tubes and centrifuged at 12 000 *g* for 10 min at 4 °C. The pellets were resuspended in Nuclei Extraction Buffer 2 and centrifuged at 12 000 *g* through a layer of Nuclei Extraction Buffer 3, which contained 1.7 M sucrose, 10 mM Tris-HCl, pH 8.0, 2 mM MgCl_2_, 0.15% Triton X-100, 5 mM β-mercaptoethanol, 0.1 mM PMSF, and protease inhibitors, in microfuge tubes for 60 min at 4 °C. The nuclear pellets were lysed in Nuclei Lysis Buffer, which contained 50 mM Tris-HCl, pH8.0, 10 mM EDTA, 1% SDS, and protease inhibitors. The lysed nuclei were sonicated four times with a Bioruptor (UCD-200) in a water bath at 4 °C, with each sonication period consisting of 15 s on and 15 s off for 5 min and followed by centrifugation. The clear supernatants, which contained the sonicated chromatin, were transferred to new tubes. Immunoprecipitation (IP) was performed using 5 μl of chromatin, and the following IP steps were conducted using the Magna ChIP™ HiSens Kit. Aliquots of the dilution were used for the IP assays. The anti-GCN5 antibody was generated using two synthetic peptides (H2N-CARGADTDSDPDESED and H2N-SSRNTKLKTESSTVKLC); both peptide epitopes are located between amino acids 85 to 99 and between amino acids 136 to 150, which is the N-terminal region of the protein, provided by Prof. D-X Zhou, and the specificity of this GCN5 antibody was confirmed by protein gel blots (Benhamed *et al.*, 2006, 2008). The anti-H3K14ac and anti-H3K9ac antibodies were purchased from Upstate Biotechnology. *CHALCONE SYNTHASE* (*CHS*) and *AT4G03800* (gypsy-like retrotransposon family gene) were amplified as endogenous controls for the anti-GCN5 and anti-H3K9/H3K14 antibodies, respectively. The immunoprecipitated DNA was analysed by quantitative PCR in three biological replicates using the primer sets listed in Supplementary Table S4. Amplified DNA from the chromatin fractions prior to antibody incubation were used as the controls (inputs). The fold-enrichment was normalized to the chromatin inputs.

### GUS histochemical and fluorometric assays

Three homologous transgenic T3 lines of *ProCER3*::*GUS*/Ws and the corresponding homologous *ProCER3*::*GUS*/*gcn5-2* lines were used for GUS histochemical analysis. The seedlings were grown under strictly identical conditions. After pollination, Arabidopsis stems, siliques, flowers, and young leaves were vacuum-infiltrated with staining buffer (2 mM potassium ferricyanide, 10 mM phosphate buffer, 0.5% Triton X-100, and 1 mg ml^−1^ X-Gluc) and then incubated overnight. The tissue was then incubated in ethanol and acetic acid (1:1) for 4–8 h and cleared in 80% ethanol. The samples were observed with a stereomicroscope (Olympus SEX16).

For the quantification of GUS activity, we used the fluorometric assay based on the method of Jefferson *et al.* (1987). Total protein extracts from stems of three independent lines for each construct (*ProCER3*::*GUS*/Ws and *ProCER3*::*GUS*/*gcn5-2*) were determined using bovine serum albumin (BSA) as a standard according to the Bradford assay (Bradford, 1976). Fluorescence was measured using 4-methylumbelliferone (4-MU) as a substrate, with an excitation wavelength of 365 nm and an emission wavelength of 455 nm in a BioTek Synergy HT Multi-Mode Microplate Reader (BioTek, Vermont, USA). GUS activities of the extracts were calculated as nanomole 4-MU per minute per milligram protein.

Both GUS histochemical and fluorometric assays were conducted at least three times, and only the transgenic lines with stable GUS signals throughout different generations were selected for further analysis.

### Scanning electron microscopy

Six-week-old stems were attached to double-sided carbon sealing tape. The specimens were examined under a Hitachi TM3000 Tabletop Scanning Electron Microscope at 15 kV, and the digital recordings were saved as TIFF files.

### Cuticular wax analysis

Cuticular wax analysis was conducted as previously described by Chen *et al.* (2011). Because the environmental conditions could affect configuration and distribution of the surface wax structures, all the plants we used in this study were cultivated in strictly controlled temperature and humidity. Six-week-old stems were collected and pictures were taken (with an adjacent ruler) for later determination of area using ImageJ (https://imagej.nih.gov/ij/). Cuticular waxes were extracted by immersing the samples for 30 s in 1 ml of chloroform containing 10 μg of tetracosane (Fluka) as an internal standard. Three biological replicates per genotype were performed. Five individual stems were used for each replicate. The extracts were transferred to reactive vials, dried under nitrogen gas, and derivatized by adding 20 μl of N, N-bis-trimethylsilyltrifluoroacetamide (Macherey-Nagel) and 20 μl of pyridine, and incubated for 40 min at 70 °C. These derivatized samples were then analysed using a gas chromatography–flame ionization detector (GC-FID, Agilent, Technologies) and GC-MS (Agilent gas chromatograph coupled to an Agilent 5973N quadrupole mass-selective detector).

Consistent with previous studies (Haslam *et al.*, 2015; Lee and Suh, 2015; Li *et al.*, 2016), the content of the unidentified components (which showed no significant differences among genotypes) was excluded from the total wax load.

### Statistical analysis

Statistical analyses of the phenotypic data and expression levels were performed using Student’s *t*-test in Excel. To assess the overall differences in the stem cuticular wax composition among genotypes, we compared the means using one-way ANOVA together with a Bonferroni adjustment test in R.

### Accession numbers

Sequence data from this article can be found in the Arabidopsis Genome Initiative or GenBank/EMBL databases under the following accession numbers: *CER3*, AT5G57800; *GCN5*, AT3G54610; *CER26*, AT4G13840; *CER1-L1*, AT1G02190; *WSD1*, AT5G37300; *AT2*, AT5G55370; *FAR3*, AT4G33790, *ACTIN8*, AT1G49240; *CHS*, AT5G13930; AT4G03800. The RNA-seq reads used for this study are deposited at the National Center for Biotechnology Information Short Read Archive under the accession number SRP093334.

## Results

### Mutation of Arabidopsis *GCN5* impairs stem cuticular wax deposition

**Fig. 1.**
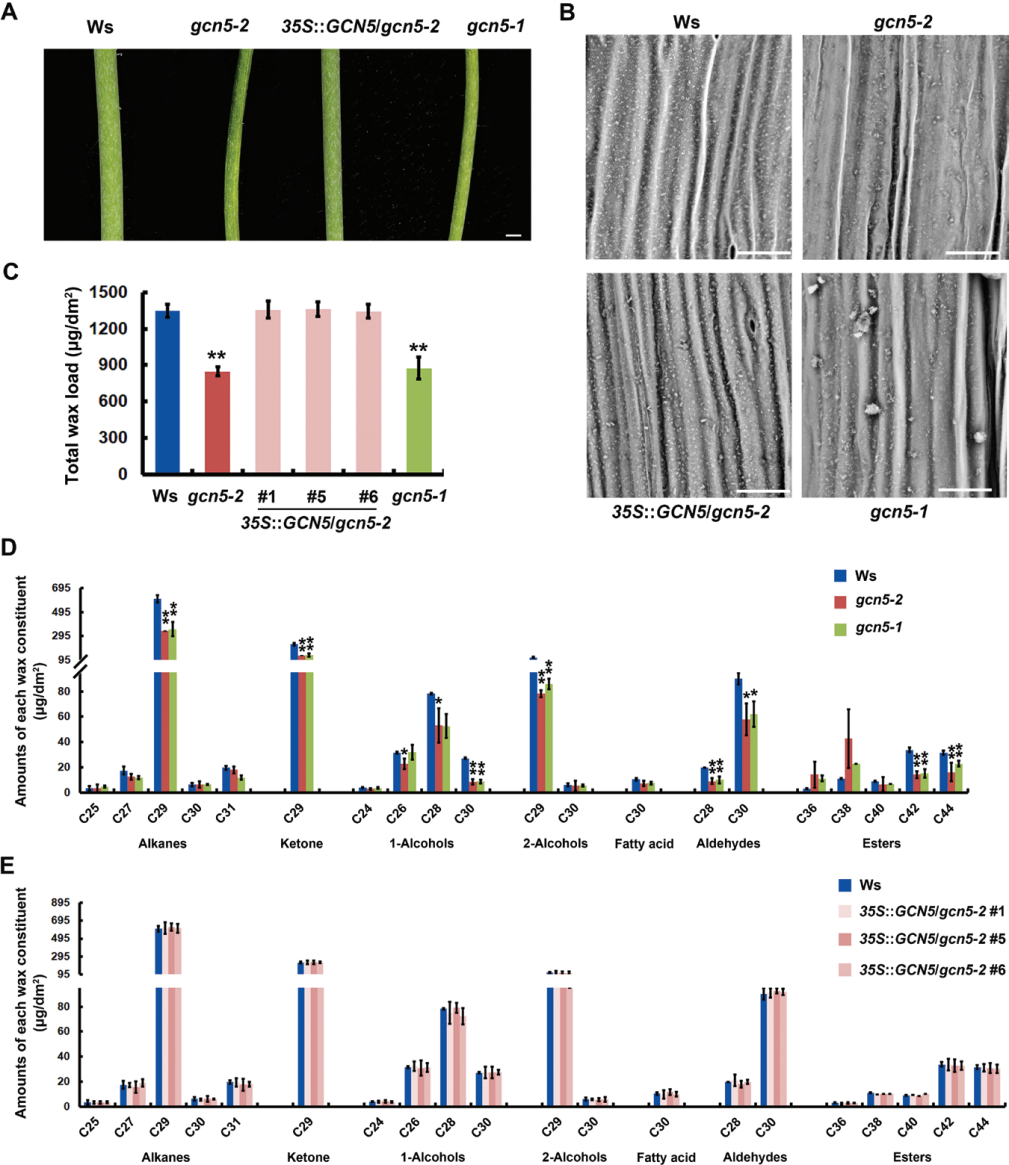
Mutations in GCN5 are responsible for defects in the stem cuticular wax in Arabidopsis. (A) Light reflectance and (B) SEM observations of 6-week-old stems from wild-type Ws, and *gcn5-2*, *35S*::*GCN5*/*gcn5-2* transgenic lines (#1, #5, #6), and *gcn5-1* mutants. Scale bars: (A) 1 mm, (B) 30 μm. (C) Total cuticular wax content, calculated per unit area of 6-week-old stems from the four different genotypes. (D) Cuticular wax composition of 6-week-old stems for wild-type Ws, and *gcn5-2* and *gcn5-1* mutants, and (E) for Ws and *35S*::*GCN5*/*gcn5-2* transgenic lines (#1, #5, #6). The chemical classes and main chain-lengths of each constituent are indicated. Data are means (±SD) of three biological replicates. *P<0.05, **P<0.01; Student’s *t*-test.

To investigate the potential roles of histone modification in cuticular wax accumulation, 23 T-DNA insertion mutants that harbor disruption in histone acetylation or methylation genes were selected for analysis, namely *ashh1*, *ashh2*, *ashh3*, *ashh4*, *ashr2*, *ashr3*, *atx1*, *atx2*, *atx4*, *atx5*, *atxr2*, *atxr3*, *atxr4*, *gcn5-2*, *hda2*, *hda2c*, *hda5*, *hda7*, *hda9*, *hda13*, *hda18*, *hda19*, and *srt2*. The stem cuticular wax of these mutants and the wild-type controls were observed and compared using SEM. As shown in Supplementary Figs S1 and S2, we found wide variations for cuticular wax crystal morphology and crystallization patterns in the mutants and wild-types. For example, *atx4*, *atxr4*, *ashr2*, and *hda9* showed more cuticular wax crystals than the wild-type (Col-0), whereas less abundant wax was observed in *atx5*, *ashh2*, and *hda18*. Remarkably, fewer stem wax crystals were observed on the surface of the *gcn5-2* mutant relative to the wild-type (Ws), which led to the appearance of a glossy stem (Fig. 1A, B). The compositions of the cuticular wax of the *gcn5-2* mutant and wild-type were quantified by GC-FID and GC-MS analysis. The total cuticular wax content in the *gcn5-2* mutant was approximately 63% of the control (Fig. 1C), which was attributable to notable decreases in the major wax constituents in the mutant stem, including alkanes (C29), ketone (C29), primary alcohols (C26, C28, and C30), secondary alcohols (C29), aldehydes (C28 and C30), and esters (C42 and C44; Fig. 1D and Table 1).

**Table 1.** Cuticular wax composition of stems in the wild-type Ws, *gcn5-2*, *gcn5-1*, and complementary transgenic lines

	Total	Alkanes	Ketones	1-Alcohols	2-Alcohols	Fatty acids	Aldehydes	Esters
Ws	1347.99 ± 54.38	650.81 ± 34.27	226.39 ± 12.94	140.44 ± 2.34	122.16 ± 6.91	10.39 ± 1.13	109.86 ± 4.70	87.93 ± 4.82
***gcn5-2***	847.49 ± 35.42**	375.94 ± 7.98**	133.64 ± 2.16**	87.01 ± 22.68*	83.59 ± 4.50**	7.03 ± 2.08	66.84 ± 14.77**	93.45 ± 38.77
***#1***	1357.28 ± 67.64	659.13 ± 34.75	230.33 ± 20.01	138.11 ± 17.45	122.64 ± 8.57	9.75 ± 3.34	111.81 ± 14.64	85.52 ± 7.39
***#5***	1360.93 ± 61.58	666.21 ± 27.19	230.35 ± 19.85	141.23 ± 15.52	116.23 ± 12.49	11.59 ± 2.21	110.60 ± 8.14	84.72 ± 17.21
***#6***	1344.06 ± 57.09	655.99 ± 27.72	228.46 ± 8.61	134.99 ± 11.51	117.54 ± 8.16	9.87 ± 1.85	111.51 ± 8.10	85.72 ± 14.12
***gcn5-1***	875.47 ± 90.61**	387.21 ± 41.12**	136.54 ± 13.30**	96.61 ± 17.24*	96.91 ± 16.31*	7.38 ± 1.03	72.00 ± 10.66**	78.82 ± 7.08

Data are means (±SD; *n*=3) for the total wax load and each constituent class in μg dm^–2^. Five individual stems were used for each replicate. #1, #5, #6 represent three complementary transgenic lines (*35S*::*GCN5*/*gcn5-2*#1, #5, #6). *P<0.05, **P<0.01; Student’s *t*-test. 1-Alcohols, primary alcohols; 2-Alcohols, secondary alcohols.

To confirm that the observed defects in stem wax were indeed caused by the disruption of GCN5, another well-established T-DNA insertion mutant allele of GCN5 (*gcn5-1*) was used for further investigation. As expected, the *gcn5-1* mutant also exhibited wax-deficient phenotypes (Fig. 1 and Table 1). Moreover, a genetic complementation experiment was performed by introducing a full-length GCN5 coding sequence driven by the CaMV35S promoter into the *gcn5-2* mutant. Given the low-fertility pollen of the *gcn5-2* mutant found in previous studies (Bertrand *et al.*, 2003; Xing *et al.*, 2015), we first generated three independent homologous *35S*::*GCN5*/Ws transgenic plants harboring significantly high GCN5 expression levels, and crossed them with *gcn5-2* mutants (Supplementary Fig. S3B). After three generations, three independent homozygous *35S*::*GCN5*/*gcn5-2* (#1, #5 and #6) transgenic lines were obtained (Supplementary Fig. S3B). No obvious phenotype differences were observed between *35S*::*GCN5*/Ws transgenic plants and the wild-type (Supplementary Fig. S3C). However, *35S*::*GCN5*/*gcn5-2* transgenic lines exhibited similar phenotypes to the wild-type (Fig. 1, Table 1, and Supplementary Fig. S3C), indicating that constitutive expression of *GCN5* could rescue the *gcn5-2* wax deficiency. These results indicated that GCN5 is essential for the normal accumulation of cuticular wax on the stem surface of Arabidopsis.

### RNA-seq analysis reveals significant alteration of lipid-related gene expression in the *gcn5-2* mutant

To investigate whether the reduction of cuticular wax content in the *gcn5-2* mutant was caused by decreased expression of wax-related genes, total RNA of 6-week-old stems of the *gcn5-2* mutant and the wild-type Ws were isolated for high-throughput RNA sequencing and transcriptomic comparison. Three biological replicates per genotype were performed and the correlation coefficients of each genotype showed favorable reproducibility (Supplementary Fig. S4). The high reliability of the RNA-seq data was verified by qPCR of 10 randomly selected genes (Supplementary Fig. S5). For each sample, values for reads per kilobase of exon model per million mapped reads (RPKM) were calculated, and the genes with at least 2-fold change and a false discovery rate value *P*≤0.05 were selected. Compared with the control, we found that 54% (2616 genes) of the total of differentially expressed genes were down-regulated in *gcn5-2* mutant stems. Because GCN5 usually positively regulates transcriptional processes (Servet *et al.*, 2010) and mutation of GCN5 decreased the total wax load in Arabidopsis stems, these 2616 genes were expected to be direct or indirect GCN5 target genes and might be involved in cuticular wax accumulation.

We then used GO analysis of the 2616 candidate genes using agriGO v2.0 (Tian *et al.*, 2017), and the categories showed considerably high enrichments in lipid metabolic process, cellular lipid metabolic process, and lipid biosynthetic process (*P*≤6.2 × 10^–6^). Moreover, a significant fraction of genes involved in metabolic and/or biosynthetic processes (including glycerolipid, neutral lipid, fatty acid, and unsaturated fatty acid), lipid catabolic process, and regulation of lipid metabolic and biosynthetic processes were enriched (Table 2), suggesting that GCN5 is involved in wax accumulation by modulating the transcription of lipid-related genes. For further screening, 145 non-redundant genes from three most-abundant lipid-related GO terms (GO:0006629, GO:0044255, and GO:0008610) were analysed together with their biological properties and functions in cuticular wax development as described in previous reports (Bernard and Joubès, 2013; Lee and Suh, 2015). Finally, we filtered five cuticular wax genes that are potential target genes of GCN5, namely *WSD1*, *CER3*, *CER26*, *CER1-L1*, and *Long-chain-alcohol O-fatty-acyltransferase 2* (*AT2*; Table 3).

**Table 2.** Lipid-related GO categories for the 2616 candidate genes down-regulated in the *gcn5-2* mutant

GO ID	Term	Query item	Query total	Bg item	Bg total	*P*-value
GO:0006629	Lipid metabolic process	145	2598	994	28362	2.7 × 10^–7^
GO:0044255	Cellular lipid metabolic process	100	2598	649	28362	2.5 × 10^–6^
GO:0008610	Lipid biosynthetic process	83	2598	522	28362	6.20 × 10^–6^
GO:0046486	Glycerolipid metabolic process	24	2598	122	28362	0.0011
GO:0045017	Glycerolipid biosynthetic process	15	2598	79	28362	0.011
GO:0046460	Neutral lipid biosynthetic process	6	2598	18	28362	0.012
GO:0006638	Neutral lipid metabolic process	7	2598	24	28362	0.013
GO:0019216	Regulation of lipid metabolic process	11	2598	54	28362	0.019
GO:0016042	Lipid catabolic process	31	2598	224	28362	0.024
GO:0046890	Regulation of lipid biosynthetic process	9	2598	43	28362	0.028
GO:0006636	Unsaturated fatty acid biosynthetic process	6	2598	24	28362	0.036
GO:0006631	Fatty acid metabolic process	33	2598	252	28362	0.037

Query item: number of down-regulated genes in the *gcn5-2* mutant annotated as the corresponding GO term. Query total: number of down-regulated genes in the *gcn5-2* mutant. Bg item: number of genes in Arabidopsis whole genome annotated as the corresponding GO term. Bg total: number of genes in Arabidopsis whole genome.

**Table 3.** Potential GCN5-regulated genes involved in cuticular wax synthesis

Gene	Name	Annotation
*AT5G37300*	*WSD1*	Bifunctional wax synthase/acyl-CoA:diacylglycerol acyltransferase
*AT5G57800*	*CER3*	ECERIFERUM 3
*AT4G13840*	*CER26*	ECERIFERUM 26
*AT1G02190*	*CER1-L1*	Protein CER1-like 1
*AT5G55370*	*AT2*	Long-chain-alcohol O-fatty-acyltransferase 2

### ChIP assays identify target genes of GCN5 involved in cuticular wax biosynthesis

**Fig. 2.**
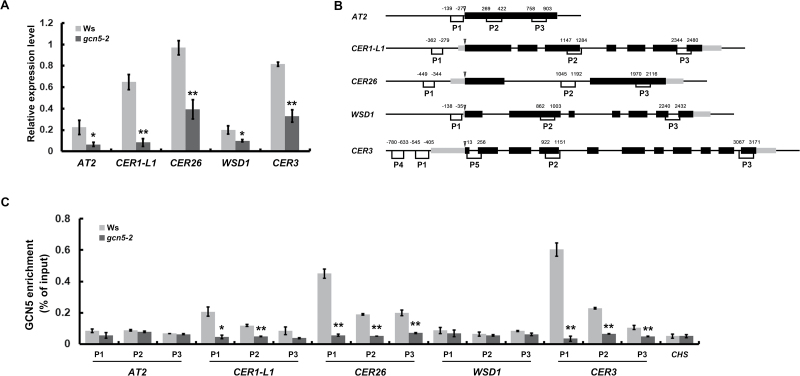
Identification of GCN5 target genes. (A) Expression levels of the GCN5 target genes as determined by qRT-PCR in 6-week-old stems of the wild-type Ws and the *gcn5-2* mutant. (B) Diagram representing the genomic structure and primer sets (indicated by P1–P5) analysed for ChIP-qPCR in the *AT2*, *CER1-L1*, *CER26*, *WSD1*, and *CER3* genes. The exact distance (in bp) of the primers from to the ATG start codon sites (indicated by triangles) are labeled. Black boxes represent exons and gray boxes represent untranslated regions (UTRs). (C) ChIP analysis with nuclei extracted from cross-linked, 6-week-old stems of Ws and the *gcn5-2* mutant and antibody-specific for GCN5. The *CHS* gene was used as a negative control, which provided background level for the ChIP samples. Data are means (±SD) from at least three biological replicates. *P<0.05, **P<0.01; Student’s *t*-test.

To confirm the GCN5-regulated target genes in stem cuticular wax biosynthesis, we first detected the transcript levels of the five candidate genes derived from RNA-seq data by qRT-PCR. As expected, their expression levels were significantly reduced in the *gcn5-2* mutant (Fig. 2A). The candidate genes were then analysed by ChIP assays, using 6-week-old stems of the *gcn5-2* and Ws plants and GCN5-specific antibodies (Benhamed *et al.*, 2006, 2008). ChIP-qPCRs with three primer pairs spanning the promoter regions and gene body regions of each gene (*WSD1*, *CER3*, *CER26*, *CER1-L1*, and *AT2*) were conducted for binding tests (Fig. 2B). *CHS* was used as the negative control as its expression is not affected by GCN5 (Benhamed *et al.*, 2006). As shown in Fig. 2C, significant decreases in enrichment in the *gcn5-2* mutant were observed for most of the examined regions of the *CER3*, *CER26*, and *CER1-L1* genes relative to the wild-type, especially in the transcription start-site region (P1) of *CER3*. By contrast, no significant changes in GCN5 enrichment were detected in any tested regions for the other two genes (*WSD1* and *AT2*).

**Fig. 3.**
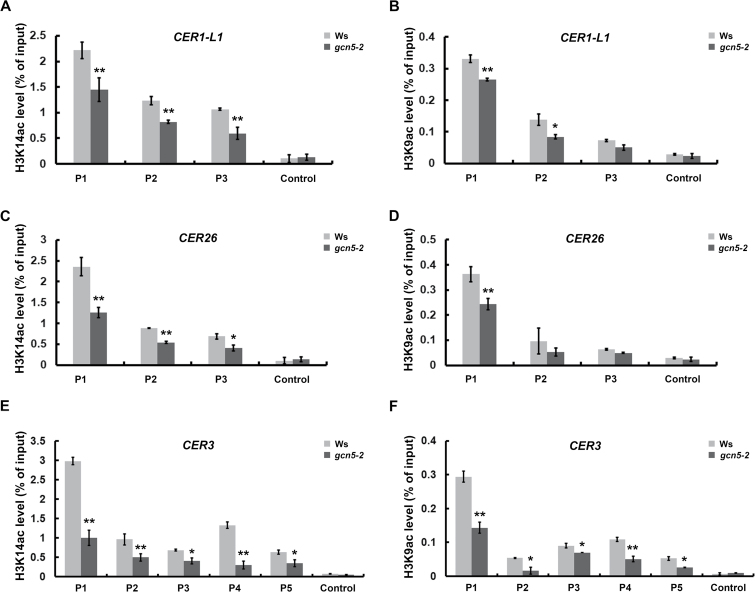
H3K14 and H3K9 acetylation levels on GCN5 target genes. ChIP analysis of H3K14 and H3K9 acetylation on (A, B) *CER1-L1*, (C, D) *CER26*, and (E, F) *CER3* genes. Nuclei extracted from cross-linked, 6-week-old stems of the wild-type Ws and *gcn5-2* mutant and antibodies specific for H3K14ac and H3K9ac. *AT4G03800* (gypsy-like retrotransposon family gene) was used as a negative control. Data are means (±SD) from at least three biological replicates. *P<0.05, **P<0.01; Student’s *t*-test.

Previous studies have reported that GCN5 is specifically responsible for H3K14 acetylation (H3K14ac) and that it influences the H3K9ac and H3K27ac at the promoters of their targets, which are positively correlated with gene expression (Bertrand *et al.*, 2003; Bhat *et al.*, 2003; Earley *et al.*, 2007). Thus, we analysed the acetylation levels of H3K14 and H3K9 at the *CER3*, *CER26*, and *CER1-L1* loci using the primer sets indicated in Fig. 2B. Consistently, both the H3K14ac and H3K9ac levels of these candidate genes were significantly decreased in the *gcn5-2* mutant compared to the wild-type, especially at the promoter regions (Fig. 3), which was in accordance with the expression profiles (Fig. 2A). Collectively, these data indicated that *CER3*, *CER26*, and *CER1-L1* are the targets of GCN5, and their expression can be regulated by GCN5 by modulating their H3K14 and H3K9 acetylation.

**Fig. 4.**
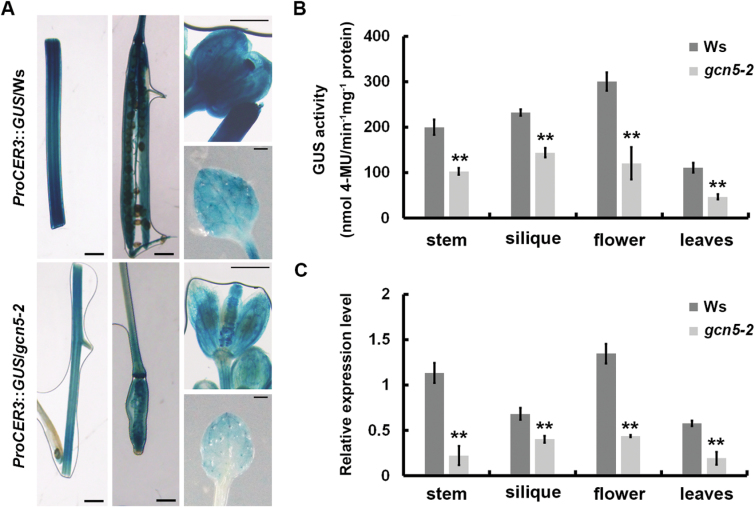
Expression patterns of *CER3* in ceriferous tissues of the wild-type Ws and *gcn5-2* mutant. (A) Spatial expression patterns of the *CER3* gene in transgenic Ws and *gcn5-2* plants harboring the *CER3* promoters fused to the GUS gene. Promoter activity was visualized through histochemical GUS-staining in stems, siliques, flowers, and young leaves of 6-week-old plants. Scale bars: stems and siliques, 1.5 mm; flowers and leaves, 2 mm. (B) Quantification of the GUS activity using 4-methylumbelliferone (4-MU) as a substrate and (C) *CER3* expression levels in stems, siliques, flowers, and young leaves of 6-week-old plants. Data are means (±SD) from three biological replicates. **P<0.01; Student’s *t*-test.

CER3 is a critical enzyme for cuticular wax synthesis (Aarts *et al.*, 1995; Chen *et al.*, 2003; Rowland *et al.*, 2007). Interestingly, the cuticular wax composition of *cer3* mutant stems was quite similar to that of the *gcn5-2* mutant stem, especially for aldehydes, alkanes, (secondary alcohols, and ketone (Lai *et al.*, 2007; Rowland *et al.*, 2007). Therefore, we hypothesized that *CER3* might be a critical target of GCN5 and decided to investigate the gene expression patterns of *CER3* in ceriferous (i.e. wax-producing) tissues of *gcn5-2* and Ws. Isogenic *ProCER3*::*GUS*/*gcn5-2* and *ProCER3*::*GUS*/Ws lines were obtained in which each transgene was homozygous and correspondingly inserted into a single genomic locus. GUS staining revealed weaker signals in stems, siliques, flowers, and young leaves of the *gcn5-2* mutant compared to that of the control (Fig. 4A). Quantification of GUS activity by fluorometric assay consistently revealed significantly lower activity in three independent homozygous *ProCER3*::*GUS*/*gcn5-2* lines than in the corresponding *ProCER3*::*GUS*/Ws lines (Fig. 4B). qRT-PCR analysis also showed that the *CER3* expression levels were decreased in different tissues of *gcn5-2* mutant as compared to the wild-type (Fig. 4C). These results provided further evidence that the expression of *CER3* is positively regulated by GCN5 in ceriferous tissues.

### Overexpression of *CER3* rescues the stem cuticular wax-deficient phenotype in the *gcn5-2* mutant

**Fig. 5.**
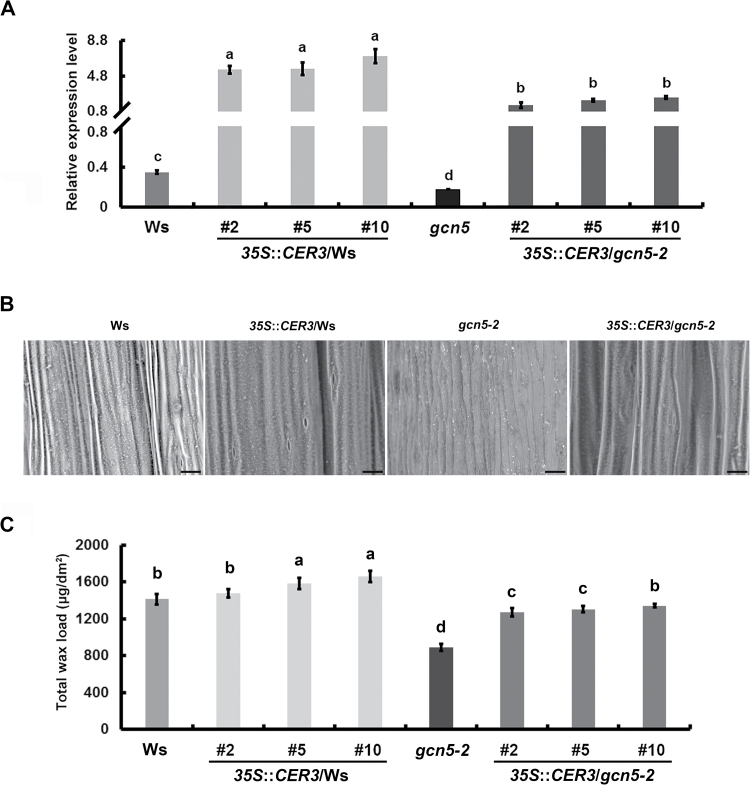
Overexpression of *CER3* in the *gcn5-2* mutant increases the cuticular wax component and restores it to the wild-type Ws level. (A) Relative expression levels of *CER3* in plants of Ws, *35S*::*CER3/*Ws (#2, #5, #10)*, gcn5-2*, and *35S*::*CER3*/*gcn5-2* (#2, #5, #10). Total RNA was isolated from 6-week-old stems of Ws and the *gcn5-2* mutant. *ACTIN8* was used as an endogenous control. Data are means (±SD) from at least three biological replicates. (B) Scanning electron microscopy of the stems of Ws, *35S*::*CER3/*Ws (#2, #5, #10), *gcn5-2*, and *35S*::*CER3*/*gcn5-2* (#2, #5, #10). Scale bars: 20 μm. (C) Total cuticular wax content was calculated over the unit area of 6-week-old stems in plants of Ws, *35S*::*CER3/*Ws (#2, #5, #10)*, gcn5-2*, and *35S*::*CER3*/*gcn5-2* (#2, #5, #10). The mean expression levels and total wax load were compared using one-way ANOVA together with a Bonferroni adjustment test in R. Different letters indicate significant differences among genotypes (*P*<0.05).

To assess the role of *CER3* in GCN5-modulated biosynthesis of stem cuticular wax, we studied the accumulation and composition of the *CER3* overexpression transgenic lines in both the Ws and *gcn5-2* mutant backgrounds. Transcript levels of the *CER3* in *35S*::*CER3*/Ws and *35S*::*CER3*/*gcn5-2* transgenic lines increased significantly compared with the controls (Fig. 5A). SEM imaging showed that the deposition of wax crystals on the *35S*::*CER3*/*gcn5-2* stem was obviously increased compared with that of the *gcn5-2* mutant and that it basically recovered to the level of Ws, but no significant differences were observed between *35S*::*CER3*/Ws and Ws (Fig. 5B).

**Fig. 6.**
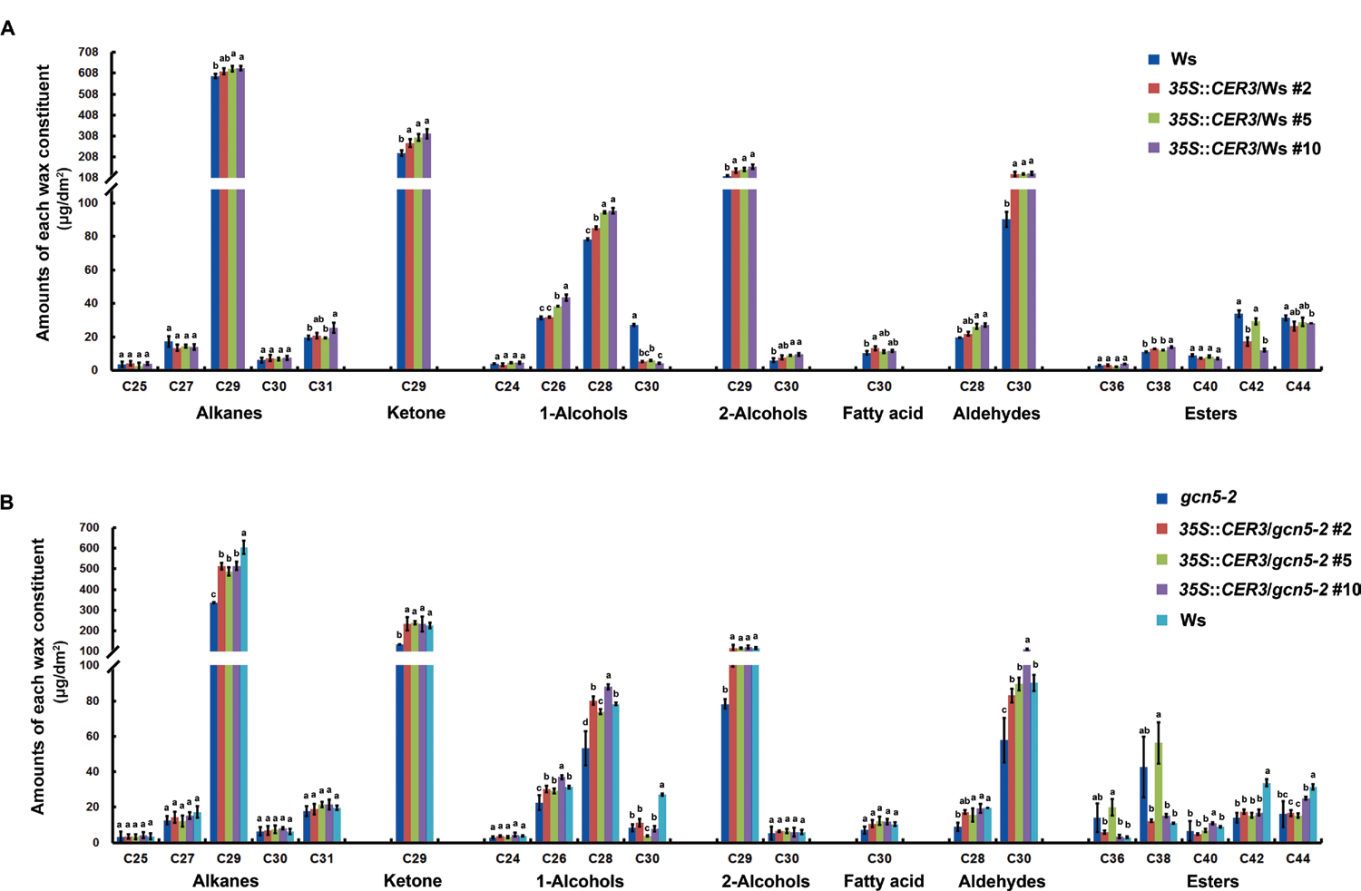
Cuticular wax composition of the stems of wild-type Ws, 35S::*CER3/*Ws (#2, #5, #10), *gcn5-2*, and 35S::*CER3*/*gcn5-2* (#2, #5, #10) from 6-week-old plants. The chemical classes and main chain-lengths of each constituent are indicated. Data are means (±SD) of three biological replicates. (A) Comparison between Ws and *35S*::*CER3/*Ws (#2, #5, #10) and (B) among Ws, *gcn5-2*, and *35S*::*CER3*/*gcn5-2* (#2, #5, #10) transgenic plants. The mean values were compared using one-way ANOVA together with a Bonferroni adjustment test in R. Different letters indicate significant differences among genotypes (*P*<0.05).

In addition, the total load and composition of stem cuticular wax of *35S*::*CER3*/Ws, *35S*::*CER3*/*gcn5*, Ws, and *gcn5-2* plants were measured by GC-FID and GC-MS analysis. Although the wax crystal loading showed little increase under SEM observation (Fig. 5B), the total amount and individual components of stem cuticular wax in two of the three *35S*::*CER3*/Ws transgenic lines (#5 and #10) were significantly increased as compared to Ws, especially for the amount of alkanes, primary alcohols, and ketone, which were consistent with the expression levels of the *CER3* gene (Figs. 5C, 6A). Notably, the total wax load in *35S*::*CER3/gcn5-2* transgenic plants was obviously increased compared with that of the *gcn5-2* mutant, and in the *35S*::*CER3/gcn5-2* #10 transgenic line it was even restored to the wild-type level (Fig. 5C). Analysis of the cuticular wax composition showed that the contents of C29 alkane, C29 ketone, C26 and C28 primary alcohols, C29 secondary alcohols, and C30 aldehyde significantly increased in the *35S*::*CER3*/*gcn5-2* transgenic lines compared with the control, thus providing genetic evidence that *CER3* plays an important role in GCN5-regulated cuticular wax biosynthesis (Fig. 6B). It should be noted that overexpression of *CER3* did not completely restore all wax component defects in the *gcn5-2* mutant, for example C30 primary alcohol and some esters, and this may be attributed to other GCN5-regulated target genes in the wax biosynthetic pathway. *FATTY ACID REDUCTASE3* (*FAR3*, also known as *CER4*), encodes an alcohol-forming fatty acyl-coenzyme A reductase, and is involved in the synthesis of primary alcohols (Rowland *et al.*, 2006; Wang *et al.*, 2015). Thus, we analysed the transcript levels of *FAR3* in *gcn5-2*, Ws, and *35S*::*CER3* transgenic plants using qRT-PCR. However, the expression levels of *FAR3* in these plants were not obviously changed (Supplementary Fig. S6).

## Discussion

Lipids are an essential constituent of all plant cells, including fatty acids (FAs), waxes, sterols, and others (Li-Beisson *et al.*, 2013). Post-translational modification of histone tails plays an important role in epigenetic regulation of gene expression, and this includes histone acetylation, methylation, phosphorylation, and ubiquitination (Pfluger and Wagner, 2007). As a member of the HAT enzymes, GCN5 is a versatile regulator of Arabidopsis development and stress responses (Bertrand *et al.*, 2003; Benhamed *et al.*, 2006; Kornet and Scheres, 2009; Hu *et al.*, 2015; Xing *et al.*, 2015). Recently, we found that GCN5 is involved in FA biosynthesis by affecting the acetylation levels of *FAD3* (Wang *et al.*, 2016). Here, we observed that GCN5 is essential for lipid metabolism in Arabidopsis stems. Firstly, mutation of *GCN5* in Arabidopsis compromised the content of multiple lipid compounds (including very-long-chain alkanes, aldehydes, ketones, and alcohols), which resulted in a complete deficiency in stem cuticular wax accumulation. This wax deficiency could be fully rescued by complementation with *35S*::*GCN5*. Secondly, GO analysis indicated that down-regulated genes in the *gcn5-2* mutant were enriched in categories related to lipid synthesis, including the lipid biosynthetic process, neutral lipid biosynthetic process, glycerolipid biosynthetic process, and unsaturated fatty acid biosynthetic process. Thirdly, ChIP assays demonstrated that *CER1-L1*, *CER26*, and *CER3*, which encode proteins involved in VLCFA production and alkane-forming pathways of wax synthesis, are target genes of GCN5. Finally, enrichment of H3K9ac and H3K14ac at the promoters of *CER1-L1*, *CER26*, and *CER3* was significantly decreased in the *gcn5-2* mutant compared with the wild-type. Collectively, our previous data (Wang *et al.*, 2016) and that from the present study have demonstrated that histone acetyltransferase GCN5 is involved in multiple lipid metabolic processes, from upstream *de novo* FA synthesis to subsequent wax production.

To determine the underlying mechanisms of GCN5-regulated biosynthesis of stem cuticular wax, three GCN5 target genes, *CER3*, *CER26*, and *CER1-L1* were identified. CER26 is involved in the elongation of VLCFAs (from 30 C to 32 C) and has high specificity of tissue and substrate (Haslam *et al.*, 2015; Pascal *et al.*, 2013). Although little is known about CER1-L1 other than that it is a homolog of CER1 (Bernard *et al.*, 2012), there is the possibility that, like CER1, it can physically interact with CER3 during the very-long-chain alkane biosynthesis process and contribute to the total wax load. Previous studies have reported that CER3, which catalyses redox-dependent alkane formation, is the key wax biosynthetic enzyme (Aarts *et al.*, 1995; Chen *et al.*, 2003; Rowland *et al.*, 2007). The wax components of the Arabidopsis *cer3* mutant are lacking in aldehydes, alkanes, secondary alcohols, and ketones in the stem compared with the wild-type (Rowland *et al.*, 2007). Interestingly, our GC-FID and GC-MS analyses found significant reductions in total wax in the *gcn5-2* mutant stem, especially for C30 aldehyde, C29 alkane, C29 ketone, and C29 secondary alcohols. Moreover, overexpression of *CER3* in the *gcn5-2* background significantly increased the levels of C29 ketone, C30 aldehydes, C29 alkanes, and C29 secondary alcohols, indicating that *CER3* played a pivotal role in GCN5-regulated biosynthesis of stem cuticular wax. However, we cannot rule out the possibility that other unknown genes may also contribute, which may prove an interesting area for further investigation. For example, the ester component, which cannot be catalysed by CER3, also changed significantly in the *gcn5-2* mutant stem. The *gcn5-2* mutant deficiency of C30 primary alcohols was not rescued by overexpression of *CER3*. In addition, the expression of *FAR3*, an important enzyme in primary alcohol formation, was not regulated by GCN5.

Many reports have demonstrated that acetylation of histone tails induces the accessibility of transcription factors to the nucleosomal DNA, which subsequently influences the gene expression (e.g. Lee *et al.*, 1993). As a key wax biosynthetic enzyme gene, *CER3* is regulated by both transcription factors and epigenetic modulators (Lee and Suh, 2015). *CER3* is positively regulated by two MYB transcription factors, MYB96 and MYB30, in response to drought and pathogen attack, respectively (Lee and Suh, 2013; Lee *et al.*, 2016*b*). Based on our RNA-seq data (see Supplementary Table S2), the expression of *MYB96* was up-regulated in the *gcn5-2* mutant, suggesting that it does not contribute much to the changed expression of *CER3* in the stem of this mutant. However, we cannot rule out the possibility that *CER3* could be regulated by other unknown transcription factors that are deregulated in the *gcn5-2* mutant. Recent studies showed that epigenetic modulators, namely the histone methyl transferases SDG8 (ASHH2) and SDG25 (ATXR7), were involved in wax accumulation through histone lysine methylation and/or indirectly through H2B ubiquitination by targeting *CER3*, and this was associated with diminished accumulation of lipids (Lee *et al.*, 2016*a*). Here, our observations also showed that the *ashh2* mutant exhibited mildly reduced wax crystal accumulation compared with Col-0. It might be of interest to further examine the relationships between GCN5, SDG8 (ASHH2), and SDG25 (ATXR7) in cuticular wax biosynthesis.

**Fig. 7.**
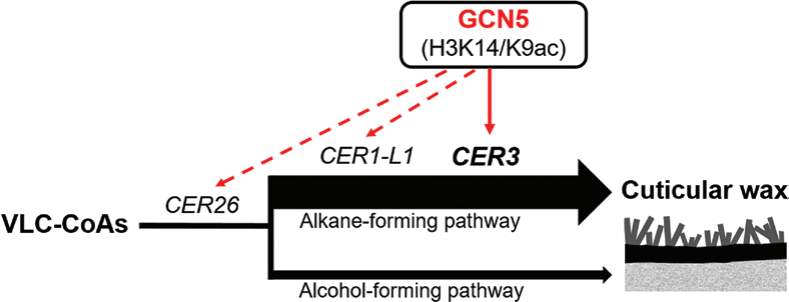
A model for the regulation of stem cuticular wax synthesis by GCN5-associated acetylation in Arabidopsis. As the wax precursors, the very-long-chain acyl-CoAs (VLC-CoA) can be processed through the alcohol-forming pathway and alkane-forming pathway, which yield 17~18% and 80% of the total cuticular wax mixture, respectively. Solid black arrows represent the cuticular wax biosynthesis pathway. The arrows from GCN5 indicate positive transcriptional regulation by GCN5 via H3K9/14ac modifications. GCN5 targets are marked at the positions where the enzymes they encode are required. CER3 is a key cuticular wax biosynthetic enzyme that catalyses the alkane-forming pathway in Arabidopsis stems (solid arrow), whereas CER26 and CER1-L1 might partially contribute to the total wax load but they were not verified functionally in this study (dashed arrows).

In conclusion, our results have demonstrated that, like histone ubiquitination and methylation, histone acetylation is also involved in the regulation of biosynthesis of stem cuticular wax, and we propose a working model to explain this process in Arabidopsis (Fig. 7). Briefly, the histone acetyltransferase GCN5 regulates the biosynthesis of stem cuticular wax by regulating the expression of *CER3*, *CER1-L1*, and *CER26* via histone acetylation at the H3K9/14 sites. Thus, interruption of GCN5 dramatically reduces the total amount of cuticular wax and changes its composition, especially with regards to alkanes, aldehydes, and ketone, which are mainly synthesized in the alkane-forming process. Remarkably, overexpression of *CER3* in the *gcn5-2* mutant could rescue the cuticular wax deficiency, suggesting that it has an important role in GCN5-mediated cuticular wax biosynthesis. Our findings provide an insight into the epigenetic regulation of cuticular wax development through histone acetylation, which may contribute to wax-related stress responses in plants.

## Supplementary data

Supplementary data are available at *JXB* online.

Table S1. Summary of the RNA-seq data and read mapping.

Table S2. Differently expressed genes in the RNA-seq data.

Table S3. GO analysis of the 2616 down-regulated genes in the stems of the *gcn5-2* mutant compared with the wild-type Ws.

Table S4. Gene-specific primer pairs in this study.

Fig. S1. Stem cuticular wax phenotype of the histone-acetylation mutants.

Fig. S2. Stem cuticular wax phenotype of the histone-methylation mutants.

Fig. S3. *GCN5* expression levels and phenotypes of the independent homologous *35S*::*GCN5* transgenic lines.

Fig. S4. Reproducibility of the RNA-seq biological replicates.

Fig. S5. Expression levels of 10 genes randomly selected to validate the accuracy of the RNA-seq data using qRT-PCR.

Fig. S6. *FAR3* expression levels in wild-type Ws, *gcn5-2*, and *35S*::*CER3* transgenic lines.

Supplementary Figures and Tables

Supplementary Table S2

Supplementary Table S3

## Abbreviations:

GCN5: general control non-repressed protein5
CER3: eceriferum3
VLCFA: very-long-chain fatty acid
HAT: histone acetyltransferase
HDAC: histone deacetylase
GO: gene ontology
GUS: β-glucuronidase
GC-FID: gas chromatography–flame ionization detector.
