# Supplementary material for: GCN5 contributes to stem cuticular wax biosynthesis by histone acetylation of CER3 in Arabidopsis
Source: J Exp Bot. 2018 Mar 1;69(12):2911–22. doi: 10.1093/jxb/ery077 (PMC5972625; doi:10.1093/jxb/ery077)
Supplement: Supplementary Figures and Tables [file ery077_suppl_supplementary_figures_and_tables.pdf]

Table S1. Summary of the RNA-Seq data and read mapping

Libraries	No. of Clean Reads	% of totally mapped reads	% of uniquely mapped reads
Ws-1	21,969,710	92.47%	84.17%
Ws-2	22,775,768	96.42%	84.16%
Ws-3	21,754,432	92.60%	83.83%
gcn5-1	21,800,486	88.64%	78.43%
gcn5-2	19,883,978	89.19%	77.00%
gcn5-3	26,895,162	87.28%	79.36%

Table S2. Differently expressed genes in the RNA-Seq data (refer to the Excel data).

Table S3. GO analysis of the 2616 down-regulated genes in the stems of *gcn5-2* mutant compared with the Ws (refer to the Excel data).

Table S4. Gene-specific primer pairs in this study

Name	sequence (5'-3')	
CER3-qF	GAGGCTCCTGTTGAGTTCCA	**Quantitative PCR analysis**
CER3-qR	GCTTGCATCTCCTTTCACCT	
GCN5-qF	GAGGGTAGAGTCGGAACAGT	
GCN5-qR	GCTCCAGTATACATGGGCCT	
AT1G01120-F	CGAAGCTAAGGGTCGGGTTA	
AT1G01120-R	AAACCGGTCGTAACGCTTTC	
AT3G28910-F	GTTTGAGGATTCTGGTGGACA	
AT3G28910-R	AGCGAGTTAATTTGGAAAACCC	
AT2G32720-F	TCGTACCCCTTGCCATTCTT	
AT2G32720-R	TGGGAAGAGACAATAACAAAGGG	
AT2G47240-F	ACATCAAAGCGGTGACAGTG	
AT2G47240-R	GAGGCCAATTTGCGGTACAT	
AT5G62470-F	TGCAAATGTCTCTTGATGCTCA	
AT5G62470-R	ACCGCATGTGAAATAGAATGC	
AT5G43760-F	GGATGACGTTGAACCGGTTT	
AT5G43760-R	ACCCGATCCAAAAGCAATCTG	
AT3G55360-F	ATTGGGCTCTTGGAAAGCAC	
AT3G55360-R	AGGAATGGAGGAAGTATCACCC	
AT5G04210-F	CAGGACATCCGAGACCATTT	
AT5G04210-R	TAACCCTCTGGCCTTTGATG	
AT1G23140-F	TAATACCAGCGACCCTTTCG	
AT1G23140-R	TCAATTCATCGTCCCACTCA	
AT2G19990-F	GACCATTCGGCGAGAATCTA	
AT2G19990-R	CCCGGAGGATCATAGCTACA	
WSD1-F	GGAAGTGCATTAGTTGGAGCA	
WSD1-R	CCCCTTGTGTGGCAGATTTG	
CER26-F	GAAAGTGATGGGGAAGTCGC	
CER26-R	TAACCGTCACAACCCTCTCC	
CER1-L1-F	CACTTGAGGGTTGGGAGGAG	
CER1-L1-R	GATGGTGGGAGAGGTTGGAA	
AT2-F	CTCGGGTCTTGCAATTACGG	
AT2-R	ACGTGCCTTTTAACAACGTCA	
FAR3-F	TTTCGATCCAAAGGTTCTCG	
FAR3-R	GGCGTTGAAGAAAATTACATCC	
ACTIN8-F	TGCAGACCGTATGAGCAAAG	
ACTIN8-R	CCGTCATGGAAACGATGTCT	
GCN5-F	TCTAGAATGGACTCTCACTCTTCCC	**Plasmid constructions**
GCN5-R	CCCGGGTATTGAGATTTAGCACCAGAT	
CER3-F	GCTCTAGAATGGTTGCTTTTTTATCAGCTT	
CER3-R	CGGGGTACCTCAATTTGTGAGTGAAGAAACAGC	
CER3-P-F	CGGGGTACCTTGTTCGCTTCACTTTGGTTT	
CER3-P-R	GCTCTAGAAATCTGAAGAAAGAGGATGGAG	

CER3-cF1	CCTCAAAACATTCCTCAGCAG	**ChIP analysis**
CER3-cR1	AGCCTCGAGCTCTGTTTCCT	
CER3-cF2	GCGATCTACGGTTACGCTGT	
CER3-cR2	CCTGTGAAGCCCCAATCTAA	
CER3-cF3	TGGCAGTACACGATGGAGAG	
CER3-cR3	ACATCAATGGCTCCAACCTC	
CER3-cF4	TTTGGCTGGAGCAAAACTAAA	
CER3-cR4	CAGAAATAAAATCTGACTGTTTCAGTG	
CER3-cF5	GCCACATAAAACACAACACCA	
CER3-cR5	GCTTTCTTCTTGTGGGAGTCA	
WSD1-cF1	CCGACGAAGATAGCAACGAC	
WSD1-cR1	GATTCTCCAACCTTTGTAGTGGA	
WSD1-cF2	GGTTGTTTGTTGGGTTTTGG	
WSD1-cR2	TATCCGATGGACAACCTTCC	
WSD1-cF3	CGCAGGAAGTGCATTAGTTG	
WSD1-cR3	TCTTGAATCGTTGTTGTGTCAA	
CER26-cF1	AGCAGAAGAAACGGGTGAGA	
CER26-cR1	TGTCCACTTCTGCGTCATCT	
CER26-cF2	CATCCCAATATGCAGATCCA	
CER26-cR2	TGCCGGTACATCACTCTGAA	
CER26-cF3	AGAGTTGCGATGGGAATTTG	
CER26-cR3	GCGTAAACAACCACCAATCC	
CER1-L1-cF1	TTCATTAAATGGGCGGCGTT	
CER1-L1-cR1	AATGGGCATTGATTCGCCTT	
CER1-L1-cF2	TTCATGTGGCCCTTCACTCT	
CER1-L1-cR2	TGCATGCGTGTAAAATGCTT	
CER1-L1-cF3	CCCAAAACATCGACTCCTGT	
CER1-L1-cR3	ATGCACTCATCACCCTCCTC	
AT2-cF1	AGCAGAAGAAACGGGTGAGA	
AT2-cR1	TGTCCACTTCTGCGTCATCT	
AT2-cF2	TCTCACCCGTTTCTTCTGCT	
AT2-cR2	ACCGTGATAAACCGGAATCA	
AT2-cF3	TGTTATCCGCAAATCTCCAA	
AT2-cR3	CAACCAGCTGTAACGAGCAA	
AtCHS-F	CACAGAAAAGGGGGCTAACA	
AtCHS-R	AGAGTTTGATGTTGCTGTTGTG	
AT4G03800-F	GAATATGCGAGGGGTCAAAA	
AT4G03800-R	CGCGGGTCATATCCTAGTTC	
HygB-F	ACATTGTTGGAGCCGAAATC	**Transgenic plant**
HygB-R	GTGTCACGTTGCAAGACCTG	**examination**

Fig. S1

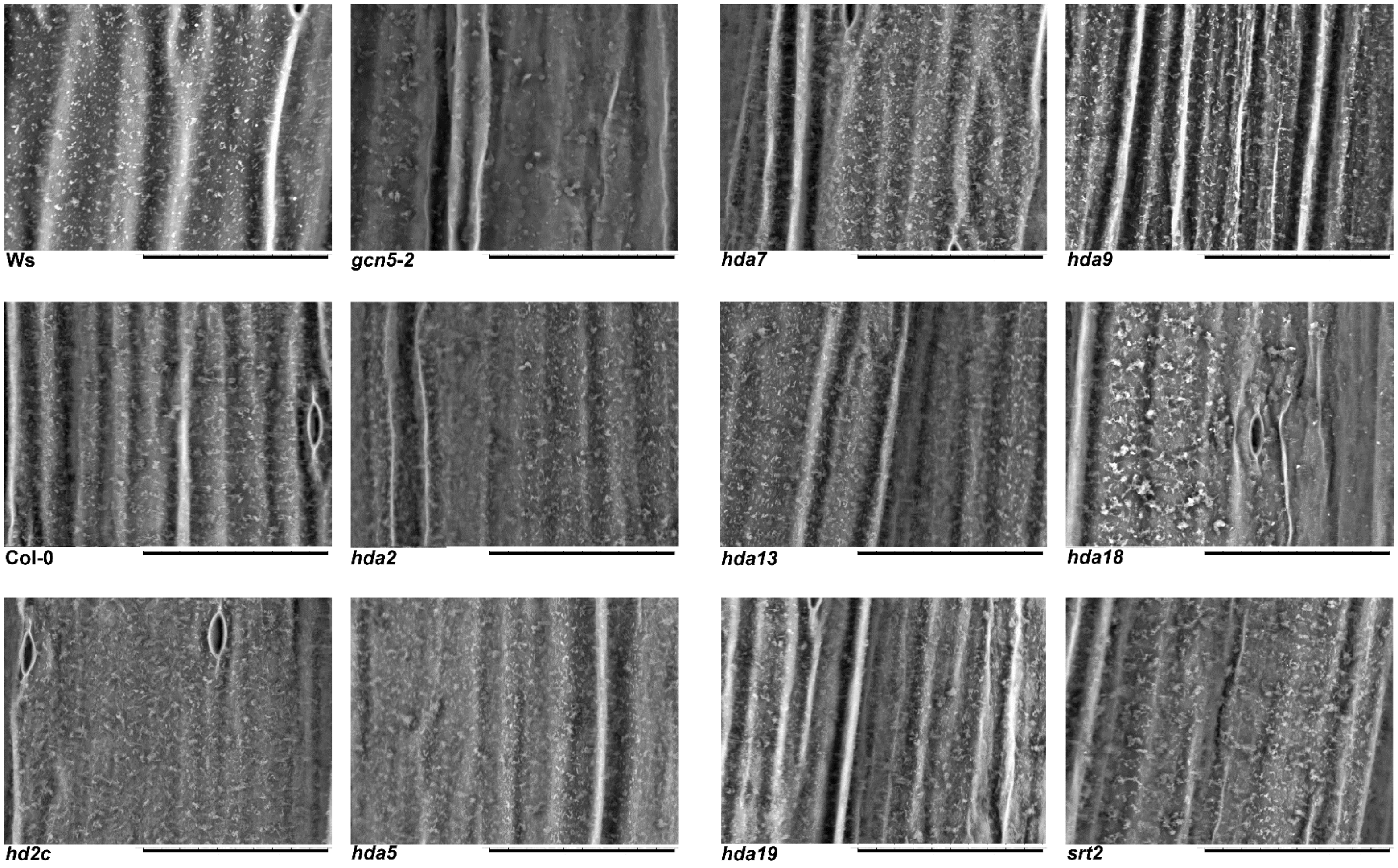

Fig. S1. Stem cuticular wax phenotype of the histone acetylation mutants

SEM images of the stems of histone acetylation mutants and wild types. Scale bars: 50 μm.

Fig. S2

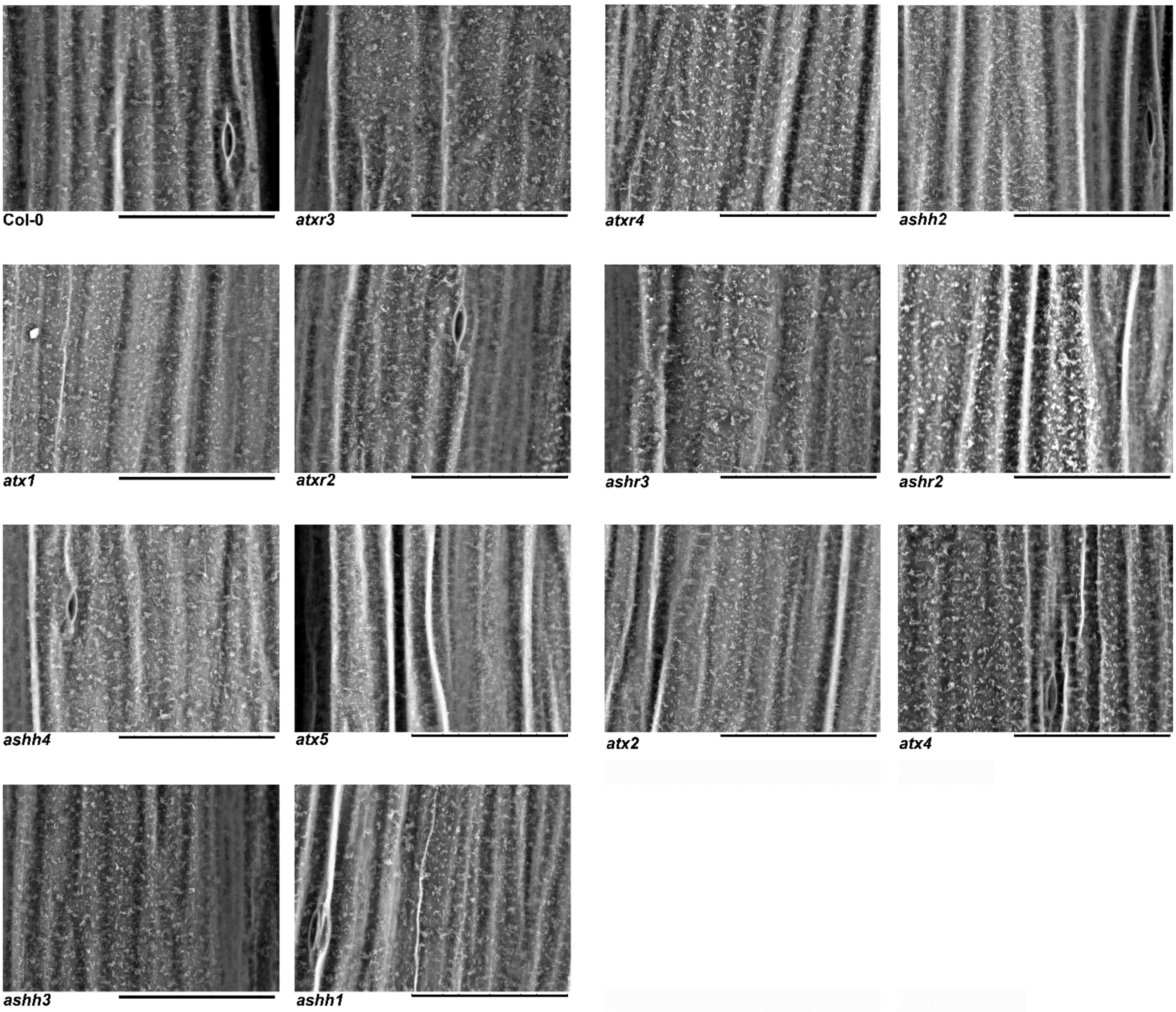

Figure S2. Stem cuticular wax phenotype of the histone methylation mutants

SEM images of the stems of histone methylation mutants and Col-0. Scale bars: 50 µm.

Fig. S3

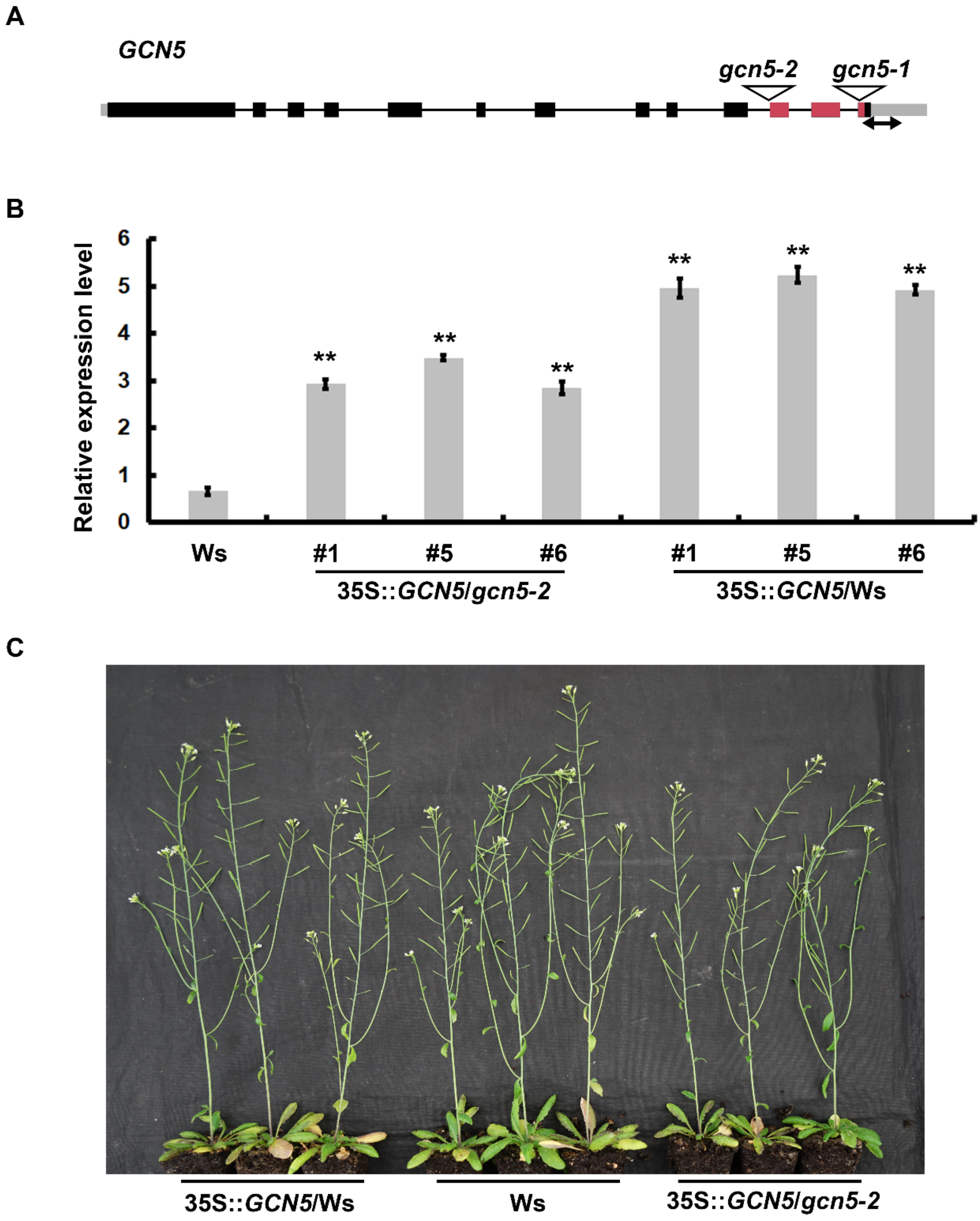

Figure S3. *GCN5* expression levels and phenotypes of the independence homologous 35S::*GCN5* transgenic lines

(A) Diagram representing the genomic structure of *GCN5*. The red rectangle represents exons encoding the bromodomain. Primer set (arrow heads) used for qRT-PCR is indicated. (B) Relative expression levels of *GCN5* in Ws, 35S::*GCN5*/Ws (#1, #5 and #6) and 35S::*GCN5*/*gcn5-2* (#1, #5 and #6) plants. Total RNA was isolated from six-week-old *Arabidopsis* stems. *ACTIN8* was used as an endogenous control. The error bars represent SD values from at least 3 biological replicates. The asterisks indicate significant differences compared to Ws (**P < 0.01; Student's t-test). (C) Phenotypes of Ws, 35S::*GCN5*/Ws (#1, #5 and #6) and 35S::*GCN5*/*gcn5-2* (#1, #5 and #6) plants at the age of six weeks.

Fig. S4

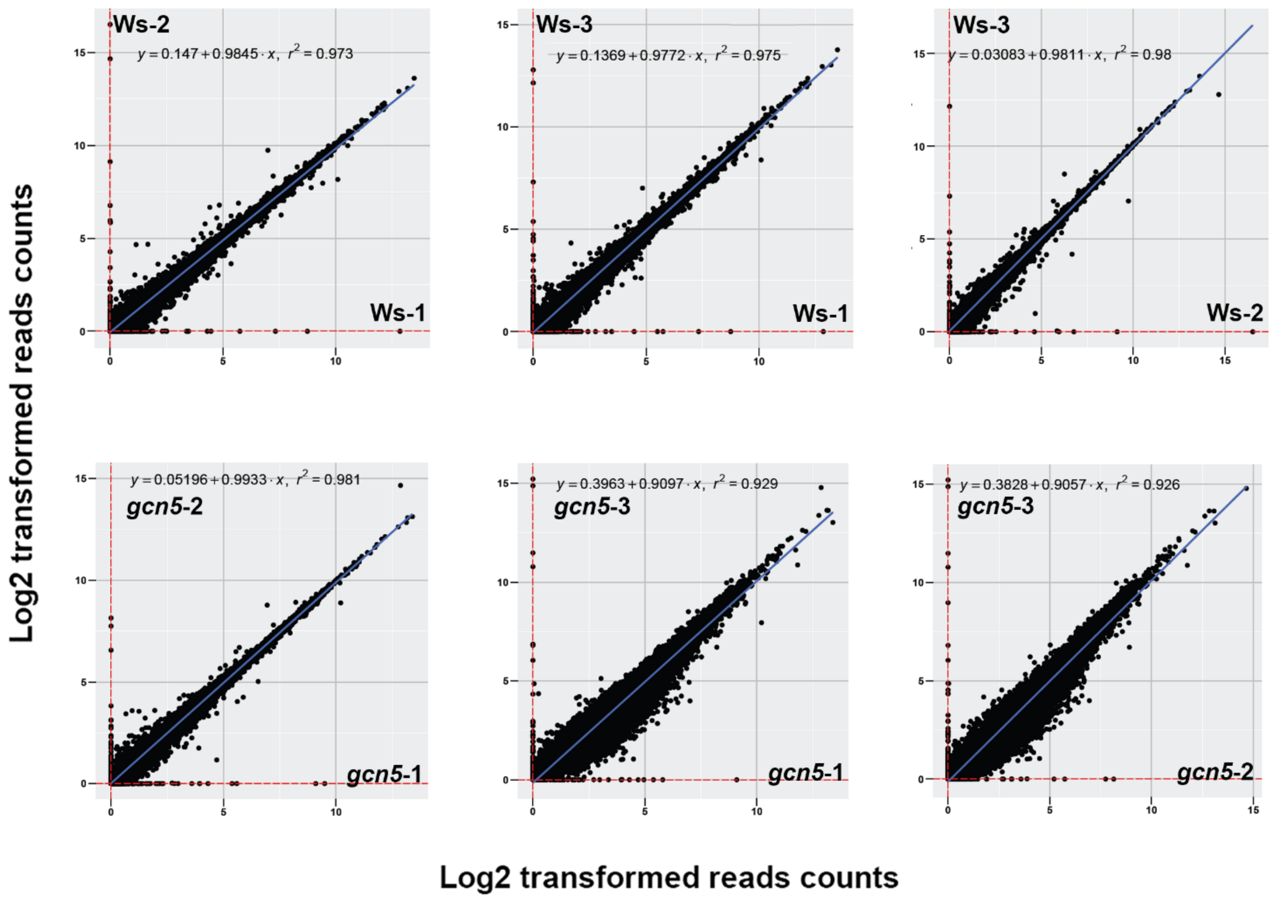

Figure S4. Reproducibility of the RNA-Seq biological replicates

High correlations between the three biological replicates of the Ws and *gcn5-2* mutants were shown. The normalized reads of three biological replicates mRNA-seq data for expressed *Arabidopsis* gene models were compared in Log2 transformed.

Fig. S5

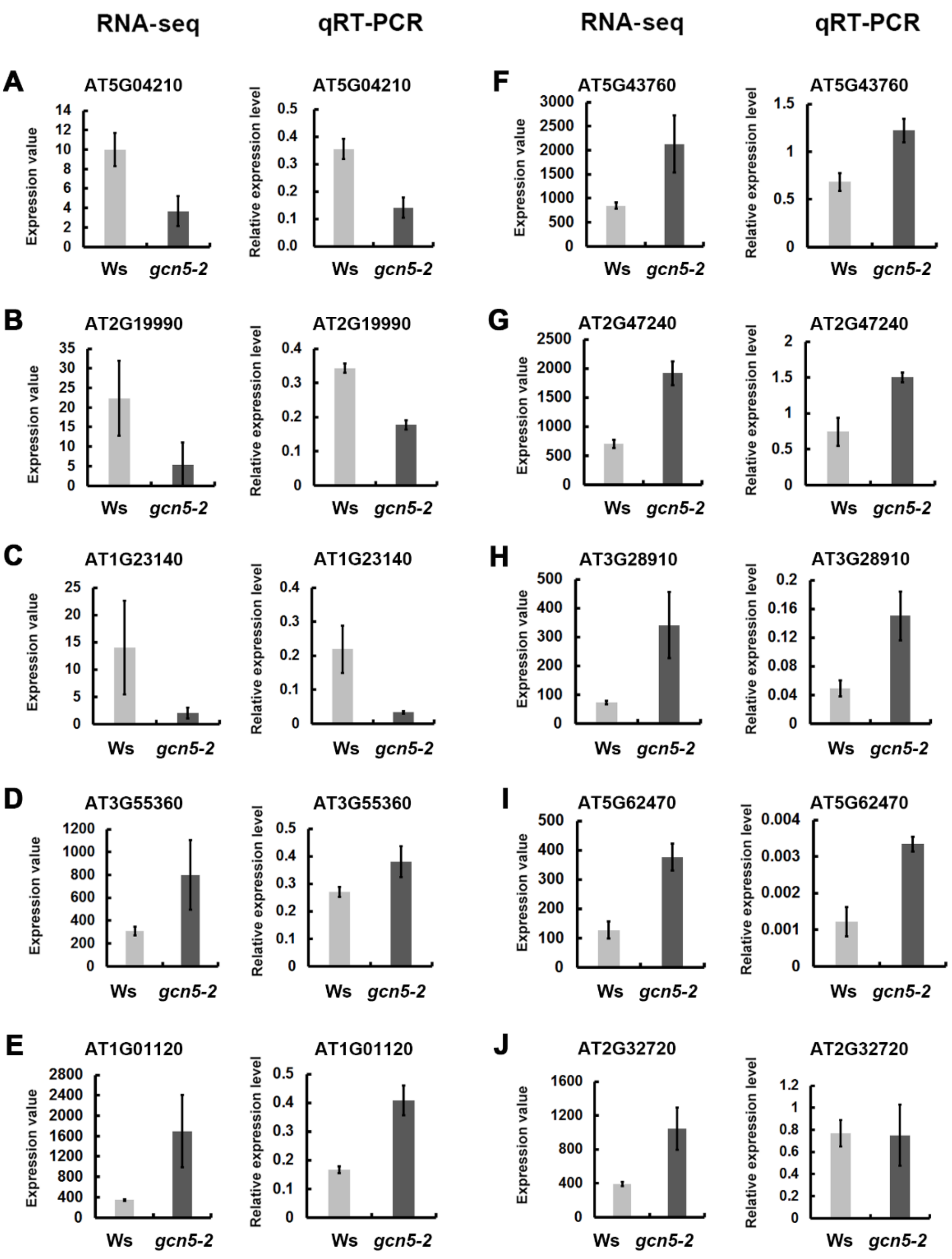

Figure S5. Ten genes randomly selected to validate the accuracy of the RNA-Seq data using qRT-PCR

Normalized expression values (RNA-seq data, left) and relative expression levels (qRT-PCR, right) of 10 randomly selected genes in the RNA-seq data. A-I, The expression patterns of nine genes based on RNA-seq were consistent with the expression patterns based on the PCR results. J, The qRT-PCR results for one genes was not exactly consistent with the expression trends in RNA-seq data. In the qRT-PCR analysis, *ACTIN8* was used as an endogenous control. The values are presented as the means and SD of three biological replicates. The accession number and gene names are listed as followed: AT2G19990, *PR-1-LIKE*; AT1G23140, *CAR8*; AT3G55360, *CER10*; AT1G01120, *KCS1*; AT5G43760, *KCS20*; AT2G47240, *LACS1*; AT3G28910, *MYB30*; AT5G62470, *MYB96*; AT2G32720, *CYTB5-B*; and AT5G04210.

Fig. S6

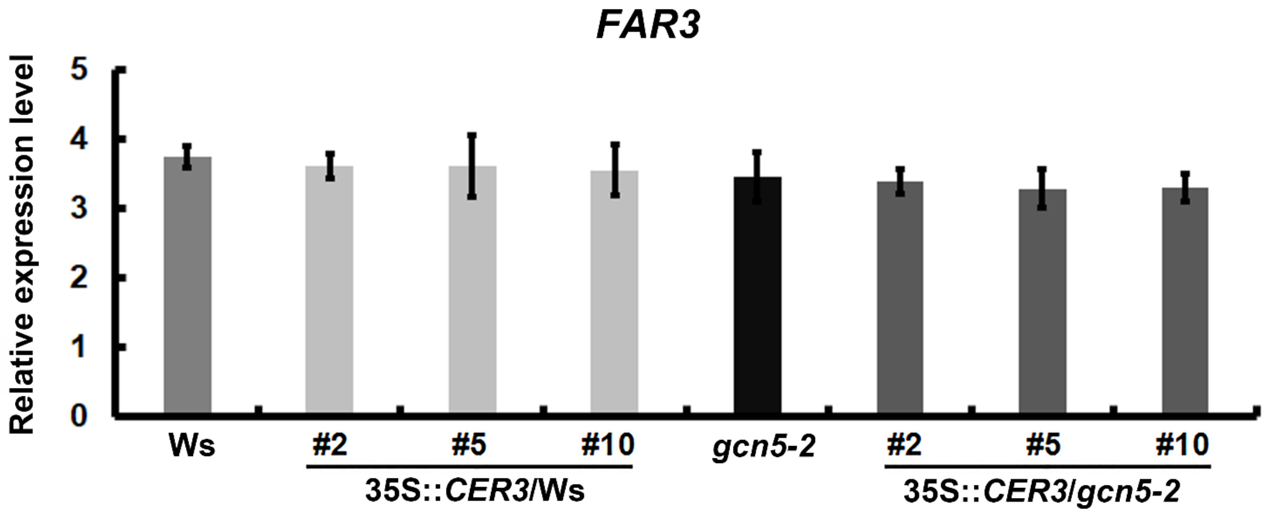

Figure S6. *FAR3* expression levels in Ws, *gcn5-2* and 35S::*CER3* transgenic lines

Relative expression levels of *FAR3* in Ws, *gcn5-2* and 35S::*CER3* transgenic plants. Total RNA was isolated from six-week-old *Arabidopsis* stems. *ACTIN8* was used as an endogenous control. The error bars represent SD values from at least 3 biological replicates.
